# Herpes Zoster in Children in the Post-Vaccination Era: Age Shift, Clinical Characteristics, and Changing Dermatomal Patterns

**DOI:** 10.3390/vaccines14010034

**Published:** 2025-12-27

**Authors:** Meryem Sena Sepetci, Nur Cihan Cosansu, Berna Solak

**Affiliations:** 1 Department of Dermatology, Sakarya Training and Research Hospital, 54100 Sakarya, Türkiye; 2 Private Clinic, Dermatology and Cosmetology, Bakırköy, 34140 İstanbul, Türkiye; 3 Department of Dermatology, School of Medicine, Sakarya University, 54290 Sakarya, Türkiye

**Keywords:** herpes zoster, pediatric dermatology, postherpetic neuralgia, vaccination, varicella

## Abstract

Background: Universal varicella vaccination has fundamentally altered the epidemiology of varicella-zoster virus (VZV) infections. While the incidence of primary varicella has declined, the characteristics of herpes zoster (HZ) in the post-vaccine generation remain a subject of debate. This study aimed to analyze the demographic and clinical characteristics of pediatric HZ in the context of a mature national immunization program. Methods: This retrospective observational study included children under 18 years of age diagnosed with HZ between 2018 and 2025 at the dermatology outpatient clinic of a tertiary hospital. Demographic, clinical, and epidemiologic variables were retrieved from hospital records. Results: A total of 64 pediatric patients (mean age: 10.5 ± 4.9 years; 59.4% male) were analyzed. The majority (93.8%) were vaccinated. Vaccinated children presented at a significantly younger age (mean: 9.1 ± 5.4 years) than those with a history of natural varicella infection without vaccination (mean: 12.3 ± 3.5 years; *p* = 0.007). The thoracic (39.1%) and cervical (35.9%) dermatomes were most frequently affected. Cervical involvement was significantly more frequent in younger children (mean age 7.1 years), whereas thoracic and lumbar dermatomes predominated in adolescents (mean age > 12 years; *p* = 0.001). Seasonal distribution was even, but the annual frequency peaked in 2023, whereas 2021 showed the lowest number. Only one patient (1.6%) developed postherpetic neuralgia, and no other complications were observed. Conclusions: Universal vaccination is associated with a shift in the onset of pediatric herpes zoster to younger individuals, often presenting with cervical dermatomal involvement hypothesized to be linked to vaccination injection sites. Clinicians should recognize that while HZ in vaccinated children is typically a self-limiting event that does not require an extensive immunologic workup in otherwise healthy, immunocompetent children, rare complications like postherpetic neuralgia can still occur. Limitations of this sin-gle-center, retrospective study include a high exclusion rate due to missing data, a small number of unvaccinated comparators, and the lack of viral genotyping to differentiate wild-type from vaccine-strain virus.

## 1. Introduction

Herpes zoster (HZ), resulting from the reactivation of latent varicella-zoster virus (VZV), is historically regarded as a disease of older adults but acts as a significant epidemiological marker when occurring in childhood. Although pediatric HZ typically follows a benign course, its occurrence in the modern era raises critical questions regarding early viral latency mechanisms, host immunity duration, and the long-term implications of universal varicella vaccination [1].

In the pre-vaccine era, primary varicella infection was nearly universal during early childhood. Epidemiological data consistently showed that the infection was most common between 2 and 8 years of age, with a peak incidence occurring at 4–6 years [2,3].

The immunological mechanism behind the shifting epidemiology involves the interplay between exogenous boosting and waning immunity [4,5]. In the absence of circulating wild-type VZV, the population-level immunity relies heavily on vaccine-induced protection, which may wane over time, potentially leading to breakthrough varicella or early-onset zoster. Furthermore, the vaccine strain itself (vOka) has the potential to establish latency in dorsal root ganglia and reactivate, albeit at a lower frequency than the wild-type virus [1,6]. Understanding whether the current pediatric HZ cases are caused by the reactivation of the vaccine strain or breakthrough wild-type infection is crucial for evaluating the long-term success of immunization programs.

Following the introduction of routine varicella vaccination in the National Immunization Program of Türkiye in January 2013, the overall incidence of primary varicella has markedly declined. This success, however, has led to a shift in viral exposure patterns and the emergence of herpes zoster cases in vaccinated children [7,8]. This epidemiologic transition has prompted ongoing debate regarding the clinical characteristics, severity, and risk factors of zoster in the post-vaccine generation compared to historical cohorts [9].

The present study aimed to analyze the demographic and clinical characteristics of children under 18 years of age diagnosed with herpes zoster, to evaluate potential risk factors, and to explore the relationships between age, vaccination status, prior varicella infection, dermatomal distribution, and complications. By examining these associations, this study seeks to provide insights into the evolving epidemiology of pediatric HZ in the context of a mature national varicella immunization program.

## 2. Methods

This retrospective observational study included pediatric patients (<18 years old) who were diagnosed with herpes zoster in the dermatology outpatient clinic between January 2018 and April 2025. Patient data were retrieved from the hospital electronic medical record system. The diagnosis of herpes zoster was made clinically based on the presence of typical grouped vesicular eruptions on an erythematous base along a dermatomal distribution, supported by medical documentation.

In Türkiye, the National Immunization Program introduced a single dose of the live attenuated varicella vaccine (Oka strain) for all children at 12 months of age starting in January 2013. Catch-up vaccination was not routinely implemented for older cohorts during the initial rollout. Our hospital serves as a major tertiary referral center in the Marmara region, providing care to a diverse pediatric population. For this study, ‘vaccinated’ status was defined as having received at least one dose of the varicella vaccine documented in the vaccination card or electronic health records. ‘Unvaccinated’ status implied no record of vaccination and a history of natural infection or no immunity. We excluded patients with equivocal diagnosis or those whose vaccination records could not be verified despite parental recall.

The recorded variables included demographic characteristics (age, sex), clinical findings (affected dermatome, predominant symptom, season of onset), history of varicella infection, varicella vaccination status, use of immunosuppressive medications, presence of comorbidities, possible triggering factors, and HZ-related complications (e.g., postherpetic neuralgia). Seasonal and annual distributions were determined according to the date of disease onset.

Only patients with complete clinical information were included in the analysis; those with missing or incomplete records were excluded.

The study was conducted in accordance with the principles of the Declaration of Helsinki and approved by the Sakarya University Faculty of Medicine Health Sciences Scientific Research Ethics Committee (approval number: E-43012747-050.04-459180-52).

## 3. Statistical Analysis

Continuous variables were expressed as mean ± standard deviation (SD) or median (interquartile range) as appropriate, while categorical variables were presented as frequencies and percentages. Normality of continuous data was assessed using the Kolmogorov-Smirnov test. Comparisons of continuous variables between groups were performed using the independent samples t-test or Mann–Whitney U test, depending on data distribution. Differences among multiple groups (e.g., age according to affected dermatome) were evaluated with Kruskal-Wallis Test followed by Bonferroni post-hoc comparisons. Associations between categorical variables (such as vaccination status, history of varicella infection, and presence of complications) were analyzed using the Chi-square test or Fisher’s exact test. Seasonal and annual case distributions were evaluated using the Chi-square goodness-of-fit test to assess deviation from uniformity. Statistical analyses and figure generation were performed using the Python programming language (version 3.9.13), utilizing the pandas (v1.4.4), scipy (v1.9.1), and statsmodels (v0.13.2) libraries. A two-tailed *p*-value < 0.05 was considered statistically significant for all analyses.

## 4. Results

### 4.1. Patients’ Characteristics

A total of 104 pediatric patients with herpes zoster were identified, of whom 64 had complete clinical records and were included in the final analysis. The exclusion of patients was strictly due to incomplete archival records regarding vaccination dates or specific clinical details. These exclusions were considered random and likely did not introduce a systematic bias regarding disease severity or clinical outcomes. The mean age of the cohort was 10.5 ± 4.9 years (range, 2–17 years), with a median age of 12 years. Among the patients, 38 (59.4%) were male and 26 (40.6%) were female. Detailed demographic and clinical characteristics are summarized in Table 1.

### 4.2. Risk Factors

A history of varicella infection was present in 27 (42.2%) patients, whereas 37 (57.8%) had no prior history of varicella. Most patients (60; 93.8%) had received the varicella vaccine, while only 4 (6.2%) were unvaccinated. Use of immunosuppressive medication was rare, identified in 2 (3.1%) patients. Regarding potential triggering factors, parent-reported emotional stress was the most frequently reported (*n* = 42; 65.6%), followed by febrile illness (*n* = 10; 15.6%) and other physical stressors such as surgery or chemotherapy.

### 4.3. Clinical Characteristics

The most commonly affected dermatome was thoracic (25; 39.1%), followed by cervical (23; 35.9%), lumbar (9; 14.1%), and trigeminal (7; 10.9%) involvement. The predominant presenting symptoms were pain (29; 45.3%), itching (23; 35.9%), and burning sensation (10; 15.6%).

**Figure 1:**
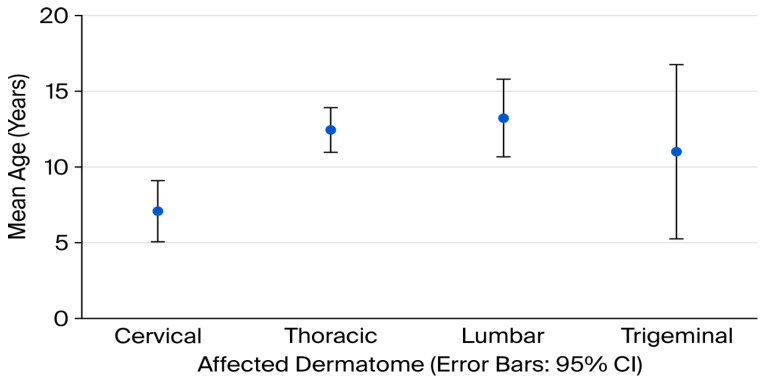
Mean age (years) according to affected dermatome in pediatric herpes zoster patients (error bar chart).

Mean age differed significantly among dermatomes (*p* = 0.001). The mean age of patients with cervical involvement was 7.1 ± 4.7 years, while those with thoracic 12.4 ± 3.6 years, lumbar 13.2 ± 3.3 years and trigeminal zoster 11.0 ± 6.2 years (Figure 1). Post-hoc Bonferroni analysis revealed that the significant differences were between the cervical and thoracic, and cervical and lumbar groups.

The mean age differed significantly between children with and without a history of varicella infection (*p* = 0.007); those with a prior infection were older (12.3 ± 3.5 years) than those without (9.1 ± 5.4 years).

A comparison of clinical characteristics according to vaccination status is presented in Table 2.

### 4.4. Seasonal and Yearly Distribution

**Figure 2:**
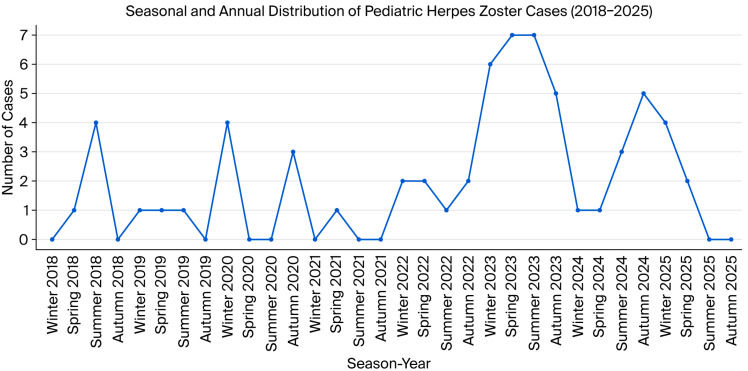
Seasonal and annual distribution of pediatric herpes zoster cases (2018–2025 (only first 4 months)).

The lowest annual frequency was observed in 2021 (*n* = 1, 1.6%), whereas the peak occurred in 2023 (*n* = 25, 39.1%), followed by a moderate decline in 2024 (*n* = 10, 15.6%). Notably, even though 2025 data covered only the first four months (until April), six cases (9.4%) had already been identified, suggesting a potentially higher annual incidence if the trend continues (Figure 2).

Herpes zoster cases were relatively evenly distributed across the seasons, with 18 (28.1%) cases in winter, 16 (25.0%) in summer, and 15 (23.4%) each in autumn and spring. No distinct seasonal clustering pattern was identified (*p* = 0.945).

### 4.5. Complications

Only one patient (1.6%) developed a complication, which was postherpetic neuralgia (PHN). This patient was a 2-year-old vaccinated girl with no history of varicella infection, no immunosuppression, and cervical dermatome involvement. Among 60 vaccinated children, 1 (1.7%) developed PHN, whereas none of the 4 unvaccinated patients did. Similarly, PHN was not observed among the 27 children with prior varicella infection, but occurred in 1 of 37 (2.7%) without such history.

Due to the very low event rate, statistical analysis of the association between previous varicella infection or vaccination and herpes zoster–related complications could not be performed. (Table 3)

## 5. Discussion

This study provides updated epidemiological and clinical data on pediatric herpes zoster (HZ) in the setting of a mature, high-coverage varicella vaccination program. Our findings demonstrate two notable trends in the post-vaccination era: (1) a younger age of onset among vaccinated children compared with those with a history of natural varicella, and (2) a predominantly mild clinical course with negligible complication rates, even in the absence of immunosuppression. These results reflect the evolving global epidemiology of pediatric HZ, where shifts in primary varicella exposure and widespread vaccine uptake have fundamentally altered the demographics and presentation of the disease.

A key observation in our cohort was that children without a history of varicella, the majority of whom were vaccinated, developed HZ at significantly younger ages than those with documented natural infection. This finding substantiates the “age-shift” hypothesis described in previous surveillance studies from the United States [8,9] and Japan [10]. The mechanism likely involves the timing of viral latency establishment: the vaccine strain (Oka) establishes latency at the time of scheduled immunization (typically ~12 months), whereas wild-type latency is acquired later in childhood, particularly in populations with high herd immunity where primary infection is delayed [6,9]. Consequently, clinical reactivation manifests at a younger chronological age in the vaccinated cohort. Crucially, this earlier onset does not correlate with increased severity; rather, it is consistently associated with a milder disease course, as confirmed in our study and previous reports [7,8,9,10].

In addition to the temporal shift, we identified a significant age-dependent variation in dermatomal distribution. Cervical zoster was significantly more frequent in younger children, whereas thoracic and lumbar dermatomes predominated in older adolescents. While definitive attribution requires viral genotyping, this distinct pattern may suggest a correlation between the site of viral entry and latency. Specifically, the predominance of cervical involvement in the younger vaccinated group likely reflects latency establishment in sensory ganglia proximal to the vaccination site, which is most commonly the deltoid region in this age group. While Civen et al. [9] reported a predilection for lumbar and sacral dermatomes attributable to thigh vaccination in infants, our findings likely reflect the establishment of latency proximal to the inoculation site, which was predominantly the deltoid region in our cohort. This hypothesis of site-dependent latency is substantiated by recent literature indicating that vaccine-associated zoster frequently follows the site of administration, such as the lumbosacral involvement observed after thigh injection [14]. Therefore, the cervical predominance in our study is consistent with the preferential use of the deltoid muscle for vaccination. Regarding cranial nerve involvement, a previous Korean study by Hwang et al. [11] reported a predominance of trigeminal involvement (32.8%) in immunocompetent children; however, their cohort consisted exclusively of hospitalized patients, who are more likely to require admission for facial zoster complications. In contrast, our outpatient-based study likely captures a broader spectrum of milder cases, revealing the prominence of cervical involvement in vaccinated children [11]. Conversely, wild-type infection, acquired via the respiratory tract, typically results in viremia and a more generalized seeding of ganglia, favoring the thoracic distribution classically seen in older children and adults [4,7,15].

Consistent with contemporary pediatric literature, HZ in our cohort was predominantly a disease of immunocompetent children [8,12]. Only two patients were receiving immunosuppressive therapy, and neither developed complications. The overall complication rate was exceptionally low, with postherpetic neuralgia (PHN) identified in only a single case (1.6%). This prevalence is markedly lower than those reported in earlier Asian cohorts, such as Japan [10] (13%) and Korea [12] (9%). However, it aligns closely with recent data from Israel [13] (<2%) and a Korean multicenter study by Hwang et al. [11], which similarly identified only one PHN case among 61 immunocompetent children. The higher complication rate reported in general hospital-based series likely reflects a selection bias toward more severe cases requiring inpatient care, whereas our outpatient-based cohort captures the predominantly mild spectrum of pediatric HZ These findings corroborate reports from the USA [9] regarding milder clinical phenotypes in vaccinated populations. Although PHN occurred in a young, vaccinated child in our study, this likely reflects individual host susceptibility rather than a systematic vaccine-related risk. Consequently, our results reinforce the clinical consensus that routine immunologic or oncologic workup is unwarranted in otherwise healthy children presenting with uncomplicated HZ [14,16].

Temporal analysis revealed a “dip-and-rebound” pattern, with a decline in cases during 2021 followed by a marked surge in 2023. This fluctuation mirrors trends observed in other infectious diseases following the COVID-19 pandemic [17]. The 2021 nadir likely resulted from reduced healthcare utilization and social restrictions. The subsequent rise in 2023 may be multifactorial, potentially attributable to the ‘immunity gap’ or ‘immune debt’ phenomenonattributable to the “immunity gap” phenomenon or a lack of “exogenous boosting” where reduced exposure to wild-type varicella in the community leads to waning cellular immunity against VZV [5]. Furthermore, the potential role of reported post-pandemic psychosocial stress as a trigger for viral reactivation cannot be over-looked, although this link requires further investigationFurthermore, the potential role of post-pandemic psychosocial stress as a trigger for viral reactivation cannot be overlooked.

Our findings have direct implications for clinical practice. The observation that pediatric HZ in the post-vaccine era presents with a milder phenotype suggests that aggressive diagnostic evaluations (such as lumbar puncture or extensive imaging) and antiviral therapies may not be necessary for the majority of immunocompetent, vaccinated children [14,16]. Clinicians should be aware of the changing dermatomal patterns, specifically the cervical predominance, to avoid misdiagnosis with other dermatological conditions. Reassurance of parents regarding the benign nature of the disease in vaccinated children is a key component of management.

To clarify the mechanisms driving the observed epidemiological shifts, the integration of molecular genotyping into routine surveillance is imperative. Distinguishing between wild-type and vaccine-strain (vOka) reactivation is not merely academic but essential for validating the ‘age shift’ hypothesis and understanding vaccine-strain latency [8,9]. Furthermore, as these vaccinated cohorts transition into adulthood, longitudinal data will be critical for assessing the durability of immunity. Evidence from such long-term monitoring will directly inform national immunization policies regarding the necessity and timing of a second dose or booster strategy to prevent waning immunity and breakthrough severe disease.

Our study has several limitations inherent to its retrospective, single-center design. First, the relatively small sample size (n = 64) and the very small number of unvaccinated comparators limit the statistical power of subgroup analyses. Second, as a tertiary re-ferral center, our study may be subject to selection bias, potentially overrepresenting atypical cases while missing mild communi-ty-managed cases; however, this is balanced by the exclusion of severe inpatients, resulting in a low complication rate. Third, the diagnosis was primarily clinical; the lack of PCR confirmation for atypical cases could introduce misdiagnosis bias. Fourth, ante-cedent factors and history of natural infection relied on parental recall, introducing potential recall bias. Additionally, potential confounding factors such as family size and daycare attendance were not systematically recorded. Finally, the lack of molecular genotyping precluded the differentiation of wild-type from vaccine-strain VZV. Therefore, our observations regarding strain-specific behavior and dermatomal patterns remain hypothesis-generating.

## 6. Conclusions

In conclusion, pediatric HZ in the post-vaccination era is characterized by an earlier age of onset but a preserved benign clinical profile. The observed prevalence of cervical dermatomal involvement in younger children provides intriguing clinical evidence hypothet-ically linking vaccination sites to latency patterns. Clinicians should be reassured that HZ in vaccinated children is a generally self-limiting event that does not, in isolation, signal underlying immunodeficiency. Continued surveillance is essential to monitor long-term epidemiologic shifts as vaccinated cohorts transition into adulthood.

## Figures and Tables

**Table 1:** Baseline Demographic and Clinical Characteristics of Pediatric Herpes Zoster Patients (*n* = 64).

Variable		*n* (%) or Mean ± SD
Sex	Male	38 (59.4%)
	Female	26 (40.6%)
Age (years)		10.5 ± 4.9
History of Varicella Infection		27 (42.2%)
Varicella Vaccination	Vaccinated	60 (93.8%)
Triggering Factor	Emotional stress	42 (65.6%)
	Febrile illness	10 (15.6%)
	Unknown	8 (12.5%)
	Surgery	2 (3.1%)
	Prematurity	1 (1.6%)
	Chemotherapy	1 (1.6%)
Affected Dermatome	Cervical	23 (35.9%)
	Thoracic	25 (39.1%)
	Lumbar	9 (14.1%)
	Trigeminal	7 (10.9%)
Comorbidity	None	59 (92.2%)
	Type 1 diabetes mellitus	2 (3.2%)
	Chronic liver disease	1 (1.6%)
	Allergy	2 (3.1%)
Complication	None	63 (98.4%)
	Postherpetic neuralgia	1 (1.6%)
Predominant Symptom	Pain	29 (45.3%)
	Itching	23 (35.9%)
	Burning sensation	10 (15.6%)
	Unable to describe	2 (3.1%)
Season of Onset	Autumn	15 (23.4%)
	Winter	18 (28.1%)
	Spring	15 (23.4%)
	Summer	16 (25.0%)

**Table 2:** Comparison of Demographic and Clinical Characteristics of Pediatric Herpes Zoster Patients According to Varicella Vaccination Status.

Variable		Unvaccinated (*n* = 4)	Vaccinated (*n* = 60)	*p*-Value
Sex	Male	1 (25.0%)	37 (61.7%)	0.295
	Female	3 (75.0%)	23 (38.3%)	
Age (years)		11.8 ± 2.2	10.4 ± 5.1	0.334
History of Varicella Infection		1 (25.0%)	26 (43.3%)	0.632
Affected Dermatome	Cervical	0	23 (38.3%)	0.337
	Thoracic	3 (75.0%)	22 (36.7%)	
	Lumbar	1 (25.0%)	8 (13.3%)	
	Trigeminal	0	7 (11.7%)	
Immunosuppressive therapy		1 (25.0%)	1 (1.7%)	0.122
Complication	None	4 (100.0%)	59 (98.3%)	>0.999
	Postherpetic neuralgia	0	1 (1.7%)	

**Table 3:** Comparison of the present study with international pediatric herpes zoster series regarding vaccination status, age of onset, and outcomes.

Country/Study	Study Period	Vaccination Policy	n (Cases)	Mean/Median Age (Vaccinated vs. Unvaccinated)	Complication Rate	Key Findings
Japan Kanamori et al., 2019 [10]	2010–2016	Mandatory (since 2014)	138	Median: 9 years	13.0%	Complications were significantly less frequent in vaccinated children (20%) compared to unvaccinated (42%). Head/neck lesions were a risk factor.
USA Weinmann et al., 2019 [8]	2003–2014	Mandatory (since 1996)	35,405	Not stratified by mean age	Not reported	Overall HZ incidence declined by 72% in the vaccinated population. Vaccinated children had a 78% lower incidence rate than unvaccinated.
Korea Hwang et al., 2019 [11]	2009–2015	Mandatory (since 2005)	126 (Total) 61 (Immunocompetent)	Median: 8.6 years (Immunocompetent group)	1.6% (PHN) (in immunocompetent group)	Only 1 PHN case occurred in 61 immunocompetent children. Trigeminal involvement was most common.
Korea Kang et al., 2021 [12]	2010–2020	Mandatory (since 2005)	602	14.0 ± 3.4	9.0%	Complications occurred even in vaccinated children, predominantly involving the head and neck.
Türkiye Gündoğdu et al., 2021 [7]	2018–2020	Mandatory (since 2013)	69	10.6 ± 4.2	1.4%	Thoracic dermatome was most common. Unvaccinated children reported significantly more pain compared to vaccinated children (*p* = 0.001).
Israel Forer et al., 2023 [13]	2000–2021	Mandatory (since 2008)	2895	8.9 ± 5.2 (Post-vac) vs. 8.9 ± 4.9 (Pre-vac)	<2.0%	HZ incidence increased after vaccination mandates, but the complication rate remained stable and low.
Türkiye Present Study, 2025	2018–2025	Mandatory (since 2013)	64	9.1 ± 5.4 (Vac) vs. 12.3 ± 3.5 (Unvac)	1.6%	Vaccinated children presented at a younger age (*p* = 0.007). Benign clinical course with negligible complications (1 case of PHN).

## Data Availability

The data that support the findings of this study are available from the corresponding author, upon reasonable request.

## References

[1] Gershon A.A., Gershon M.D., Breuer J., Levin M.J., Oaklander A.L., Griffiths P.D. (2010). Advances in the understanding of the pathogenesis and epidemiology of herpes zoster. J. Clin. Virol..

[2] Marin M., Meissner H.C., Seward J.F. (2008). Varicella prevention in the United States: A review of successes and challenges. Pediatrics.

[3] Kanra G., Tezcan S., Badur S., Turkish National Study Team (2002). Varicella seroprevalence in a random sample of the Turkish population. Vaccine.

[4] Hope-Simpson R.E. (1965). The Nature of Herpes Zoster: A Long-Term Study and a New Hypothesis. Proc. R. Soc. Med..

[5] Ogunjimi B., Van Damme P., Beutels P. (2013). Herpes Zoster Risk Reduction through Exposure to Chickenpox Patients: A Systematic Multidisciplinary Review. PLoS ONE.

[6] Gershon A.A. (2013). Varicella zoster vaccines and their implications for development of HSV vaccines. Virology.

[7] Gundogdu M., Erden N., Karagun E., Acipayam A.S.F., Vural S. (2021). Annual pattern and clinical characteristics of herpes zoster in immunocompetent children in a rural area. Dermatol. Ther..

[8] Weinmann S., Naleway A.L., Koppolu P., Baxter R., Belongia E.A., Hambidge S.J., Irving S.A., Jackson M.L., Klein N.P., Lewin B. (2019). Incidence of Herpes Zoster Among Children: 2003–2014. Pediatrics.

[9] Civen R., Chaves S.S., Jumaan A., Wu H., Mascola L., Gargiullo P., Seward J.F. (2009). The incidence and clinical characteristics of herpes zoster among children and adolescents after implementation of varicella vaccination. Pediatr. Infect. Dis. J..

[10] Kanamori K., Shoji K., Kinoshita N., Ishiguro A., Miyairi I. (2019). Complications of herpes zoster in children. Pediatr. Int..

[11] Hwang J.H., Kim K.H., Han S.B., Kim H.H., Kim J.H., Lee S.Y., Choi U.Y., Kang J.H. (2019). A clinico-epidemiological multicenter study of herpes zoster in immunocompetent and immunocompromised hospitalized children. Clin. Exp. Vaccine Res..

[12] Kang D.H., Kwak B.O., Park A.Y., Kim H.W. (2021). Clinical Manifestations of Herpes Zoster Associated with Complications in Children. Children.

[13] Forer E., Yariv A., Ostrovsky D., Horev A. (2023). The Association between Varicella Vaccination and Herpes Zoster in Children: A Semi-National Retrospective Study. J. Clin. Med..

[14] Zhang S., Kim V.H.D., Grunebaum E. (2025). Pediatric herpes zoster: Should I be concerned for immunodeficiency? A review. Front. Pediatr..

[15] Zerboni L., Sen N., Oliver S.L., Arvin A.M. (2014). Molecular mechanisms of varicella zoster virus pathogenesis. Nat. Rev. Microbiol..

[16] Gross G., Schöfer H., Wassilew S., Friese K.E., Timm A., Guthoff R., Pau H.W., Malin J.P., Wutzler P., Doerr H.W. (2003). Herpes zoster guideline of the German Dermatology Society (DDG). J. Clin. Virol..

[17] Cohen R., Ashman M., Taha M.K., Varon E., Angoulvant F., Levy C., Rybak A., Ouldali N., Guiso N., Grimprel E. (2021). Pediatric Infectious Disease Group (GPIP) position paper on the immune debt of the COVID-19 pandemic in childhood, how can we fill the immunity gap?. Infect. Dis. Now.

